# Mori Ramulus Inhibits Pancreatic β-Cell Apoptosis and Prevents Insulin Resistance by Restoring Hepatic Mitochondrial Function

**DOI:** 10.3390/antiox10060901

**Published:** 2021-06-03

**Authors:** Taewon Han, Eun Ko, Minji Kim, Moonsung Choi, Changho Lee, In-Ho Kim, Sooim Shin, Min Young Um

**Affiliations:** 1 Division of Functional Food Research, Korea Food Research Institute, Wanju 55365, Korea; korea498@kfri.re.kr (T.H.); 50036@kfri.re.kr (M.K.); chang@kfri.re.kr (C.L.); skihs@kfri.re.kr (I.-H.K.); 2 Department of Food and Biotechnology, Korea University, Sejong City 30019, Korea; 3 Department of Biotechnology & Bioengineering, College of Engineering, Chonnam National University, Gwangju 61186, Korea; ge6008@gmail.com; 4 Division of Food Biotechnology, Korea University of Science and Technology, Daejeon 34113, Korea; 5 Convergence Institute of Biomaterials and Bioengineering, Department of Optometry, College of Energy and Biotechnology, Seoul National University of Science and Technology, Seoul 01811, Korea; mschoi@seoultech.ac.kr

**Keywords:** Mori Ramulus, diabetes, pancreatic β-cells, mitochondria, apoptosis

## Abstract

Type 2 diabetes mellitus is characterized by insulin resistance and pancreatic beta (β)-cell dysfunction. Accumulating evidence suggests that mitochondrial dysfunction may cause insulin resistance in peripheral tissues. As commercial hypoglycemic drugs have side effects, it is necessary to develop safe and effective natural compound-based hypoglycemic treatments. This study aimed to investigate the hypoglycemic effects of Mori Ramulus ethanol extract (ME) in a high-fat diet (HFD)-induced diabetes mouse model to decipher the underlying mechanisms focusing on apoptosis and mitochondrial function. ME significantly decreased tunicamycin-induced apoptotic cell death and increased insulin secretion following glucose stimulation in NIT-1 pancreatic β-cells. Tunicamycin-exposed NIT-1 pancreatic β-cells showed elevated reactive oxygen species levels and reduced mitochondrial membrane potential, which were reversed by ME treatment. ME inhibited the tunicamycin-induced apoptosis cascade in tunicamycin-exposed NIT-1 pancreatic β-cells. In HFD diabetic mice, the serum-free fatty acid and insulin levels decreased following a 15-week ME administration. Glucose and insulin tolerance tests showed that ME improved insulin sensitivity. Moreover, ME ameliorated pancreatic β-cell mass loss in diabetic mice. Finally, ME-treated HFD-fed mice showed improved hepatic mitochondrial function resulting in insulin sensitivity in target tissues. Thus, ME provides protection against pancreatic β-cell apoptosis and prevents insulin resistance by improving mitochondrial function.

## 1. Introduction

Type 2 diabetes mellitus (T2DM) is a heterogeneous metabolic disorder characterized by hyperglycemia and progressive decline in insulin action, followed by beta (β)-cell dysfunction to compensate for insulin resistance [1,2,3,4]. Accumulating evidence indicates that β-cell loss results from stress responses regulated by key pathways, such as endoplasmic reticulum (ER) stress, mitochondrial dysfunction, production of reactive oxygen species (ROS), and apoptosis [5]. Given the functional importance of the ER, an organelle responsible for synthesizing, folding, and trafficking secretory and membrane proteins to the Golgi complex, the maintenance of ER homeostasis in β-cells is important [6]. Many studies have shown the correlation of ER stress-associated genes with β-cell dysfunction and apoptosis. In addition, mitochondrial dysfunction is a key player in the loss of β-cell during the evolution of T2DM [7]. This finding indicates that humans with T2DM or rodents with insulin resistance following high-fat feeding may fail to increase their mitochondrial activity [8,9]. ROS, produced by β-cell mitochondria as a result of metabolic stress, activate stress-response pathways. These alterations of mitochondrial function trigger cell apoptosis and impair glucose-stimulated insulin secretion in pancreatic β-cells [10]. Butler et al. [11] reported that T2DM subjects showed loss of β-cell volume compared with non-diabetic controls, and the frequency of β-cell apoptosis by TUNEL staining was increased in these patients. Impaired β-cell function can promote the progression of diabetes and its serious complications [12]. Therefore, protecting β-cell function can be a reasonable strategy for the treatment of T2DM.

Currently available therapeutic drugs for T2DM include insulin and various oral hypoglycemic agents, such as α-glucosidase inhibitors, sulfonylurea, metformin, biguanide, GLP-1 agonist, and SGLT-2 inhibitors [13,14]. However, as these drugs have serious side effects, the demand for new natural products with hypoglycemic activity is increasing. Recent intensive research efforts and investments have focused on the development of hypoglycemic agents using natural food sources which can reduce blood glucose levels and promote weight loss [15]. Mori Ramulus is the dry branch of *Morus alba* L. and is known as “Sangzhi” in Korea. It is a traditional medicine widely used for diabetes treatment. Some reports have demonstrated that Mori Ramulus extracts (ME) can reduce blood glucose levels in vivo [16,17,18]. The results of a recent study by Ham et al. [19] using streptozotocin-induced diabetes animal models, suggest that Mori Ramulus might be a potential hypoglycemic agent. The major active components of Mori Ramulus are mulberroside A, oxyresveratrol, resveratrol, 4-hydroxycinnamic acid, 7-hydroxycoumarin, and morin, and the components have been demonstrated to possess anti-cancer, anti-hypertensive, anti-diabetic, and anti-inflammatory properties [20]. However, it remains unclear whether Mori Ramulus is involved in modulating both pancreatic β-cell function and insulin resistance, and whether these activities are mediated by inhibiting mitochondrial dysfunction and apoptosis.

Therefore, in this study, we hypothesized that Mori Ramulus has hypoglycemic effects mediated by improving the loss of pancreatic β-cell mass and impaired insulin secretion in HFD-induced diabetes mouse model, and examined the underlying molecular mechanisms with a focus on apoptosis and mitochondrial function.

## 2. Materials and Methods

### 2.1. Sample Preparation

ME was provided by Kukjeon Pharm. Co., Ltd. (Anyang, Korea). The extracts were prepared according to a previously reported method [20]. Briefly, Mori Ramulus (*Morus alba*. L branch) was collected from *Morus alba* and dried for three days at 55 °C in a hot air dryer. The dried sample was sliced, washed, and extracted using 80% ethanol for 48 h at 85 °C in a sonicator. Then, the extracts were filtered and concentrated using a vacuum evaporator (Eyela, Tokyo, Japan), freeze-dried, and stored at −80 °C. Mori Ramulus was identified by Prof. Jeong Keum Kim at the Korea Polytechnic University. A voucher specimen was deposited at the Korea Polytechnic University.

### 2.2. High-Performance Liquid Chromatography Analysis (HPLC)

**Figure 1:**
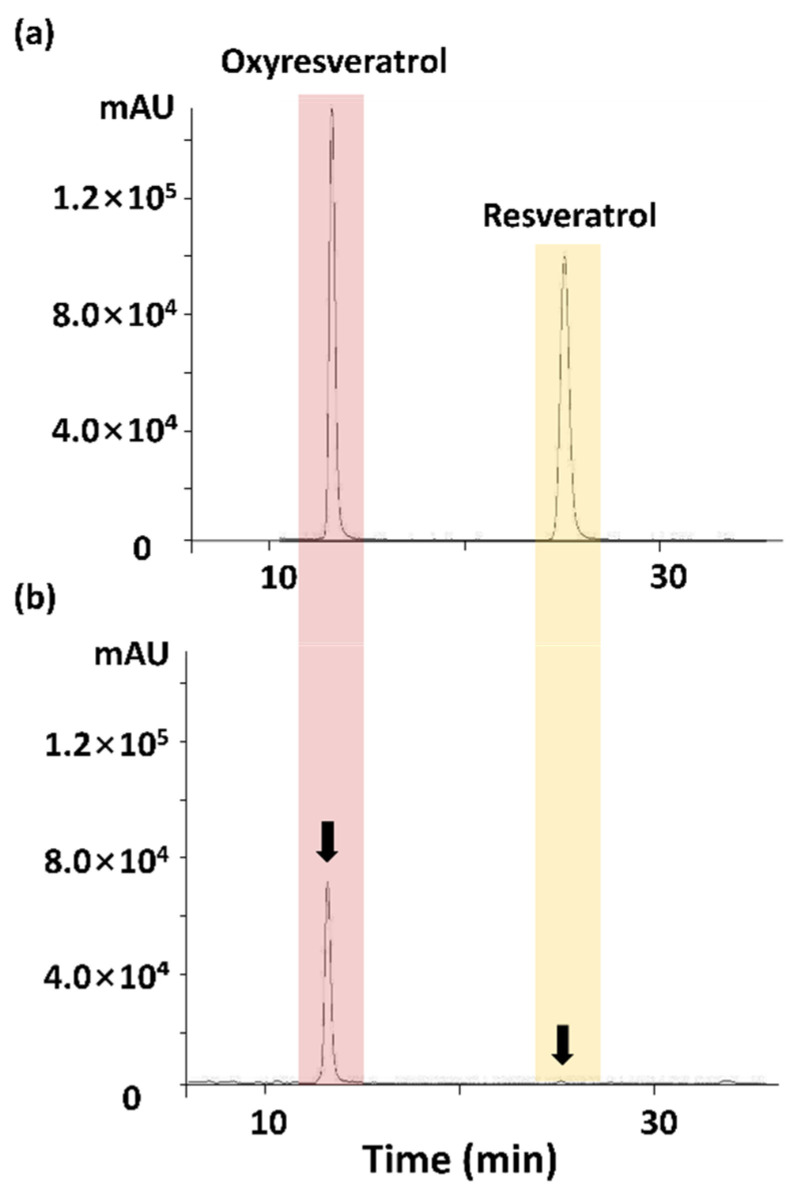
HPLC chromatogram of (**a**) standard solution and (**b**) Mori Ramulus ethanol extracts.

HPLC analysis was performed using a Jasco HPLC system (Jasco, Hachioji, Tokyo, Japan) equipped with a PU-980 pump, an AS-950-10 auto-sampler, and an MD-2010 Plus multi-wavelength detector. Chromatic separation was performed at 30 °C on a Waters Symmetry C18 5-μm column, (4.6 × 250 mm) (Waters Corporation, Milford, MA, USA). The mobile phase was 20% acetonitrile. The flow rate was 1 mL/min, and the samples were detected at UV 325 nm. The retention time of oxyresveratrol and resveratrol in ME were 13.198 and 25.135 min, respectively. The concentrations of oxyresveratrol and resveratrol in ME were found to be 385.84 μg/mg and 20.73 μg/mg of the extract, respectively (Figure 1).

### 2.3. Cell Culture and Viability

NIT-1 pancreatic β-cells (ATCC^®^, Manassas, VA, USA) were cultured in a Dulbecco’s modified Eagle medium (DMEM)-F-12 medium (Gibco, Grand Island, NY, USA) supplemented with 10% fetal bovine serum and 100 unit-100 μg/mL penicillin-streptomycin in a humidified atmosphere with 5% CO_2_ at 37 °C.

Cell viability was analyzed by the MTT [3-(4,5-dimethyl-2-thia-zolyl)-2,5-diphenyl-2-H-tetrazolium bromide] assay. The cells were exposed to the endoplasmic reticulum (ER) stress inducer tunicamycin 0.05 μg/mL (Sigma-Aldrich, St. Louis, MO, USA) for 24 h in the presence or absence of ME at various concentrations (10, 25, and 100 μg/mL). After incubation, the cells were treated with the MTT solution for 4 h. Formazan was dissolved using dimethyl sulfoxide and its concentration was determined by measuring the OD at 540 nm using a microplate reader (BioTek Instruments, Winooski, VT, USA).

### 2.4. Insulin Secretion Assay

Insulin secretion in NIT-1 pancreatic β-cells was examined according to a previously published procedure [21]. NIT-1 pancreatic β-cells were plated at a density of 4 × 10^5^ cells/well in a 48-well plate and incubated. After 24 h, cells were washed and pre-incubated with Krebs-Ringer bicarbonate (KRB) buffer containing 2.8 mM glucose for 60 min at 37 °C. Following pre-incubation, the cells were incubated with KRB buffer containing 2.8 mM glucose or 22.4 mM glucose with tunicamycin or ME for 30 min at 37 °C. The insulin amount secreted into the supernatant was determined using an insulin ELISA kit according to the manufacturer’s instructions (ALPCO, Salem, NH, USA).

### 2.5. Apoptosis Detection

Cell apoptosis was determined using a TUNEL assay kit (Abcam, Cambridge, MA, USA) in accordance with the manufacturer’s instructions. NIT-1 pancreatic β-cells were seeded in an 8-well chamber slide and the treatment was similar to that used for the cell viability assays. The nuclei were stained using 4′, 6-diamidino-2-phenylindole (DAPI). Results are expressed as the percentage of TUNEL-positive cells among the total number of cells.

### 2.6. Detection of Mitochondrial Membrane Potential

The mitochondrial membrane potential (ΔΨm) was assessed using a JC-1 fluorescence probe (Thermo Fisher Scientific, Waltham, MA, USA) according to the manufacturer’s instructions. ΔΨm depletion was observed under a Zeiss Axio Observer A1 fluorescence microscope (Zeiss, Germany), and the red/green fluorescence intensity ratio was calculated.

### 2.7. Animals and Diet

All animal experiment protocols were approved by the Institutional Animal Care and Use Committee of Korea Food Research Institute (KFRI-IACUC, KFRI-M-16037). Thirty-two C57BL/6J mice (male, 4-week-old) were purchased form Koatech (Pyeongtaek, Korea). The mice were housed in standard polycarbonate cages under controlled temperature (22–25 °C), humidity (50%), and lighting (12 h light/12 h dark cycle) conditions. After a 1-week adaption period, the mice were assigned to four groups and fed ad libitum with a normal diet (10% calories from fat, ND group), HFD (45% calories from fat, HFD group), HFD plus 0.1% ME (HFD + LS group), or HFD plus 0.25% ME (HFD + HS group) for 15 weeks. ND and HFD were obtained from Research Diets Inc. (New Brunswick, NJ, USA). Body weight was measured weekly once, and food intake was recorded 2–3 times a week throughout the study.

After feeding the mice with the experimental diets for 15 weeks, blood was collected from the abdominal aorta after overnight fasting to determine the levels of the blood biomarkers. Then, serum was separated by centrifugation at 4000 rpm for 20 min at 4 °C and stored at −80 °C for further assays. Furthermore, liver, pancreas, and adipose tissues were dissected, weighed, and stored at −80 °C until analysis.

### 2.8. Oral Glucose Tolerance Test (OGTT) and Intraperitoneal Insulin Tolerance Test (ipITT)

OGTT and ipITT were performed according to a previously published procedure [22,23], with slight modifications. Mice were fasted for 12 h with threshold glucose measurements taken at 0 min before starting the tests. The mice were then administered a glucose solution (2 g/kg body weight) intragastrically or an insulin solution (0.75 unit/kg body weight) intraperitoneally. Blood glucose levels were measured in the whole blood collected from the tail vein using a commercially available glucometer (gDoctorTM, Allmedicus Co., Ltd., Anyang, Korea) at 15, 30, 60, 90, and 120 min following administration.

### 2.9. Blood Biochemical Analysis

Serum total cholesterol (TC), HDL-cholesterol (HDL-C), triglyceride (TG), and glucose levels were determined using commercial kits (Shenyang Chemical Co., Busan, Korea). Free fatty acids (FFA) were measured in the serum using a quantification assay kit (Abcam), and insulin levels in the serum were determined using mouse ultrasensitive insulin or an adiponectin ELISA kit (ALPCO), according to the manufacturer’s instructions.

### 2.10. Histological Examination and Immunohistochemical Staining

Pancreatic tissues were fixed with 10% formalin and embedded in paraffin followed by hematoxylin and eosin (H&E) staining to evaluate the pathological changes. The stained area was observed using a light microscope (Olympus, Tokyo, Japan).

For the immunohistochemical assay, pancreatic sections were deparaffinized, rehydrated, and incubated for 30 min in 3% hydrogen peroxide containing distilled water. After washing with tris-buffered saline (TBS, pH 7.3), the sections were incubated with goat serum for 30 min. Then, the sections were incubated overnight at 4 °C with an anti-insulin antibody (1:50; BioGenex, Fremont, CA, USA). Sections were washed thrice with TBS buffer and incubated with an anti-guinea pig antibody (1:200; Vectastain, Servion, Switzerland) for 90 min at room temperature. After washing, sections were incubated with an avidin-biotin peroxidase complex (Vectastain) for 15 min and washed. Subsequently, the sections were stained with 3,3′ diaminobenzidine (DAB) and counter-stained with hematoxylin and mounted with a coverslip for microscopic evaluations. Stained sections were observed under the microscope (BX50, Olympus, Tokyo, Japan). Digital images were analyzed using the ImageJ software (National Institutes of Health, Bethesda, MD, USA).

### 2.11. Mitochondria Isolation

Liver tissues obtained from the experimental groups were homogenized. Homogenized tissues were used for mitochondrial isolation using the mitochondria isolation kit for tissues (Thermo Fisher Scientific, Waltham, MA, USA). The total mitochondrial protein amount was quantified using the protein assay dye reagent concentrate (Bio-Rad, Hercules, CA, USA).

### 2.12. Mitochondrial OXPHOS Enzyme Activity Measurement

The enzyme activities of complex I (NADH: ubiquinone oxidoreductase), complex II (succinate dehydrogenase), complex III (CoQH2-cytochrome c reductase), and complex IV (cytochrome c oxidase) were measured. Briefly, the activity of each enzyme was measured by monitoring the change in the absorption spectra of their specific substrates or products. A decrease in absorption at 600 nm as a result of dichlorophenolindophenol (DCPIP, extinction coefficient 19.1 mM^−1^cm^−1^) reduction, coupled with the reduction of decylubiquinone, was monitored for measuring complex I and II activities, and a change in absorption at 550 nm as a result of reduced cytochrome c (extinction coefficient 18.5 mM^−1^cm^−1^) by the reduction or oxidation of cytochrome c was monitored for measuring complex III and IV activities. The activity of each complex was calculated from the linear portion of the progression curve using the following Equation (1):

Enzyme activity (mol/sec∙mg) = (Δ Absorbance/sec × 1000)/[(extinction coefficient × volume of sample used in mL) × (sample protein concentration in mg/mL)](1)

### 2.13. Western Blot Analysis

NIT-1 pancreatic β-cells and liver tissues were lysed in RIPA buffer supplemented with a complete protease inhibitor cocktail (Roche Diagnostics, Mannheim, Germany). Protein concentration was determined using a BCA protein assay kit (Thermo Fisher Scientific, Waltham, MA, USA). Quantified protein samples were separated using a 10% SDS-PAGE, and then transferred onto a polyvinylidene fluoride membrane. The membranes were blocked with 5% skim milk in TBS with 0.1% Tween 20 (TBST) for 2 h, and incubated with the following specific primary antibodies overnight at 4 °C: activating transcription factor 6 (ATF6, 1:1000, Abcam), α-tubulin (1:1000, Santa Cruz Biotechnology, Dallas, TX, USA), Bax (1:1000; Novus Biologicals, Littleton, CO, USA), Bcl-2 (1:1000; Novus Biologicals), caspase-3 (1:1000; Cell Signaling Technology, Danvers, MA, USA), cleaved caspase-3 (1:1000; Cell Signaling Technology), caspase-9 (1:1000, Santa Cruz Biotechnology), cleaved caspase-9 (1:1000, Santa Cruz Biotechnology), C/EBP homologous protein (CHOP, 1:1000, Cell Signaling Technology), cytochrome C (1:1000, Santa Cruz Biotechnology), eukaryotic translation initiation factor 2A (eIF2a, 1:1000, Cell signaling Technology), inositol-requiring enzyme 1 alpha (IRE1α, 1:1000, Cell Signaling Technology), PARP (1:1000, Santa Cruz Technology), cleaved PARP (1:1000; Santa Cruz Biotechnology)), X-box-binding protein 1 (XBP-1, 1:1000, Santa Cruz Biotechnology), β-actin (1:1000; Imgenex, San Diego, CA, USA), prohibitin (1:500; Santa Cruz Biotechnology), and OxPhos Rodent WB antibody (1:250; Invitrogen, MD, USA). After washing with TBST, the membranes were incubated with horseradish peroxidase-conjugated anti-rabbit IgG secondary antibodies (1:3000, Vector, Torrance, CA, USA) for 2 h at room temperature. Immunoreactive protein bands were detected using an enhanced chemiluminescence reagent (LI-COR, Lincoln, NE, USA). Images were analyzed using the Image J software and normalized to β-actin or prohibitin levels.

### 2.14. Mitochondrial ATP Production Determination

ATP production, a primary function of mitochondria, was determined using an ATP determination kit (Thermo Fisher Scientific, Waltham, MA, USA) following a modified protocol. To measure the real-time production of ATP from isolated mitochondria, 1 mM malate, 2 mM ADP, and 1 mM pyruvate were incubated with 5 μg of mitochondria. The ATP produced was measured using the kit components according to the manufacturer’s protocol. Luminescence intensity, coupled with ATP production, was monitored using SpectraMax iD3 (Molecular Devices, San Jose, CA, USA).

### 2.15. Statistical Analyses

Results are expressed as the mean ± SD. One-way ANOVA with the post-hoc Tukey test was used for statistical analysis using SPSS 20.0 for Windows (SPSS Inc., Chicago, IL, USA). *p* value < 0.05 was considered statistically significant.

## 3. Results

### 3.1. ME Prevents Tunicamycin-Induced Cell Death in NIT-1 Pancreatic β-Cells

**Figure 2:**
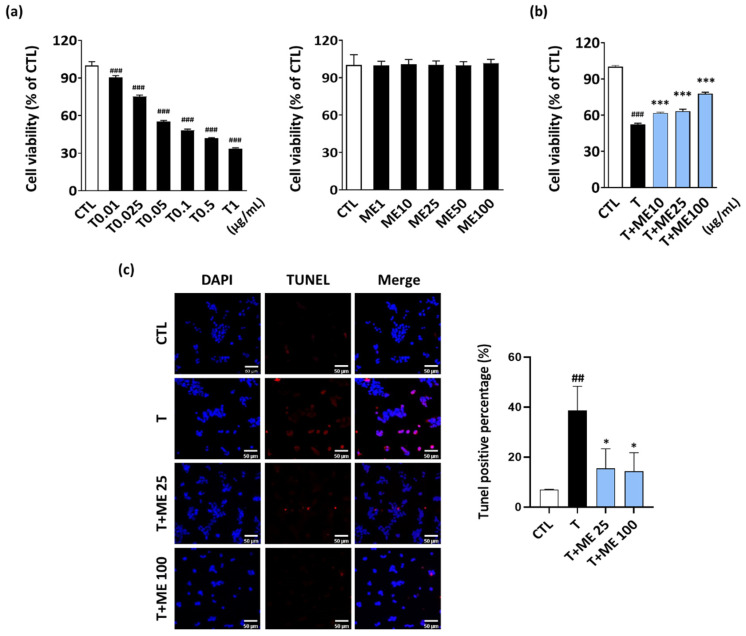
Effect of ME on the viability of tunicamycin-treated NIT-1 pancreatic β-cells. (**a**) NIT-1 pancreatic β-cells (2 × 10^4^ cells/well) were treated with tunicamycin or ME alone for 24 h. Cell viability was determined using the MTT assay. (**b**) NIT-1 pancreatic β-cells were co-treated with ME (10, 25, or 100 μg/mL) for 24 h in the absence or presence of tunicamycin (0.05 μg/mL). (**c**) In situ apoptosis was evaluated using DAPI and TUNEL staining assays. Results are the mean ± SD of three separate experiments performed in triplicate. ## *p* < 0.01, ### *p* < 0.001 versus CTL (vehicle-treated cells); * *p* < 0.05, *** *p* < 0.001 versus tunicamycin-treated cells. T: tunicamycin; ME: Mori Ramulus ethanol extract.

ER stress is well-known to induce β-cell loss and diabetes [24]. When NIT-1 pancreatic β-cells were treated with various concentrations of tunicamycin (0.01–1 μg/mL) for 24 h, cell viability was significantly decreased in a dose-dependent manner (Figure 2a), and the 50% inhibitory concentration was 0.05 μg/mL. Thus, we chose 0.05 μg/mL of tunicamycin as an optimal concentration for use in further experiments. To determine whether ME inhibits tunicamycin-induced cell death, we treated NIT-1 pancreatic β-cells with tunicamycin in the presence of various concentrations of ME. As shown in Figure 2b, ME treatment (10–100 μg/mL) effectively decreased the tunicamycin-induced cell death in a dose-dependent manner.

To investigate the protective effect of ME on tunicamycin-induced apoptosis, TUNEL staining assay was performed. The results showed that treatment of NIT-1 pancreatic β-cells with tunicamycin increased the number of apoptotic cells, compared to that in the CTL (vehicle-treated cells) group. However, ME treatment inhibited tunicamycin-induced apoptosis, although this effect was not observed in ME treatment at 10 μg/mL (data not shown). Thus, our results indicate that ME protects against tunicamycin-induced cell death in NIT-1 pancreatic β-cells.

### 3.2. ME Improves Insulin Secretion in Tunicamycin-Treated NIT-1 Pancreatic β-Cells

**Figure 3:**
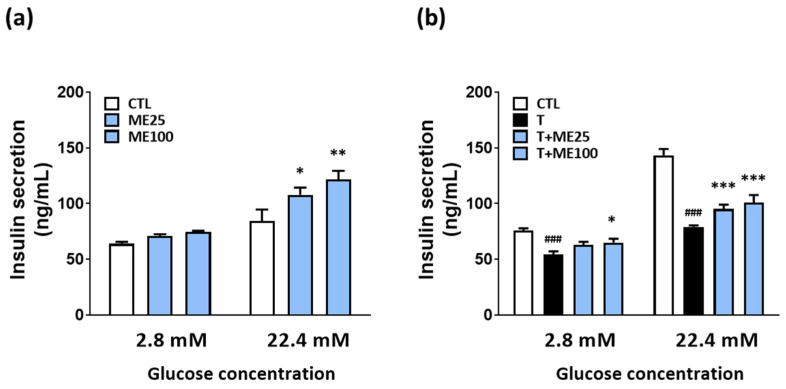
ME improved insulin secretion in NIT-1 pancreatic β-cells exposed to low or high glucose concentrations. (**a**) Treatment with ME alone in NIT-1 pancreatic β-cells. (**b**) Co-treatment of tunicamycin and ME in NIT-1 pancreatic β-cells. Results are represented as the mean ± SD of three separate experiments performed in triplicate. ### *p* < 0.001 versus CTL (vehicle-treated cells); * *p* < 0.05, ** *p* < 0.01, *** *p* < 0.001 versus tunicamycin-treated cells. T: tunicamycin; ME: Mori Ramulus ethanol extract.

We investigated the effect of ME on insulin secretion in NIT-1 pancreatic β-cells. As shown in Figure 3, treatment with ME alone at 25 and 100 μg/mL increased insulin secretion at a high-glucose concentration of 22.4 mM, but showed no changes at a low-glucose concentration of 2.8 mM. In addition, we observed a significant reduction in insulin secretion in tunicamycin-treated NIT-1 pancreatic β-cells; however, ME treatment could improve insulin secretion under both low-glucose and high-glucose stimulated conditions.

### 3.3. ME Prevents Tunicamycin-Induced ROS Formation and Activation of the Mitochondrial Apoptosis Pathway in NIT-1 Pancreatic β-Cells

**Figure 4:**
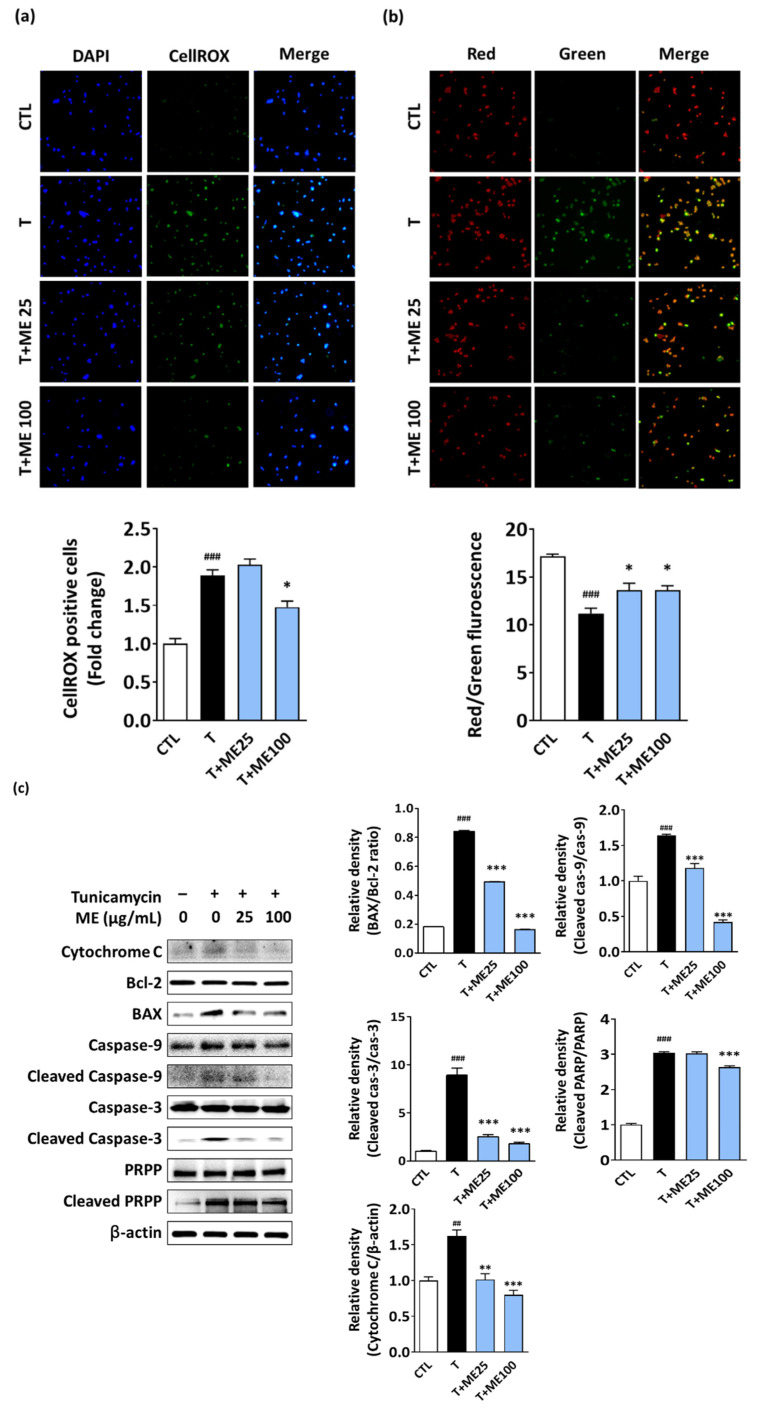
ME ameliorated ROS formation, mitochondrial dysfunction, and apoptotic signaling induced by tunicamycin treatment in NIT-1 pancreatic β-cells. (**a**) Effect of ME on the tunicamycin-induced intracellular ROS levels. Representative images of ROS staining using CellROX. (**b**) Mitochondrial membrane potential (∆Ψm) detected using JC-1 staining. Red fluorescence represents the mitochondrial aggregate form, indicating an intact ∆Ψm. Green fluorescence represents the monomeric form, indicating the dissipation of ∆Ψm. (**c**) Cell extracts were prepared 24 h after tunicamycin alone or co-treatment with ME and tunicamycin, and the levels of apoptosis-related proteins were examined using Western blotting. Results are the mean ± SD of three separate experiments performed in triplicate. ## *p* < 0.01, ### *p* < 0.001 versus CTL (vehicle-treated cells); * *p* < 0.05, ** *p* < 0.01, *** *p* < 0.001 versus tunicamycin-treated cells. ROS: reactive oxygen species; T: tunicamycin; ME: Mori Ramulus ethanol extract.

Tunicamycin-induced ER stress is known to promote ROS-mediated mitochondrial apoptosis [25]. We investigated whether ME could suppress ROS formation following exposure to tunicamycin. As shown in Figure 4a, NIT-1 pancreatic β-cells treated with tunicamycin consistently showed an approximate two-fold increase in the ROS levels compared to the CTL. However, ME and tunicamycin co-treated cells showed lower green fluorescence intensity compared to the cells treated with tunicamycin alone, clearly indicating that ME treatment suppressed the tunicamycin-induced ROS formation.

As the main reason of cellular ROS formation is mitochondrial dysfunction, we evaluated the change in ΔΨm using the JC-1 fluorescent probe (Figure 4b). Treatment with tunicamycin significantly decreased ΔΨm, while ME protected against the tunicamycin-induced decrease in ΔΨm. Similarly, using fluorescence microscopy, an increase in the red/green fluorescence ratio was observed in cells co-treated with ME and tunicamycin compared to that of cells treated with tunicamycin alone.

Considering our results, we further explored the protective mechanism of ME through the mitochondrial apoptotic pathway. Western blotting showed a notable upregulation in the expression levels of cytochrome C, BAX, cleaved caspase-9, cleaved caspase-3, and cleaved PRPP, a hallmark apoptotic execution protein, as well as downregulated expression of anti-apoptotic Bcl-2 following tunicamycin treatment for 24 h compared to the levels in the CTL group. However, these events were blocked by ME treatment in a dose-dependent manner. In addition, the increase in the BAX/Bcl-2 ratio following tunicamycin treatment was restored by ME to that of the CTL group. Thus, ME protects against tunicamycin-induced apoptotic cell death in NIT-1 pancreatic β-cells.

### 3.4. ME Improves Tunicamycin-Induced ER Stress

**Figure 5:**
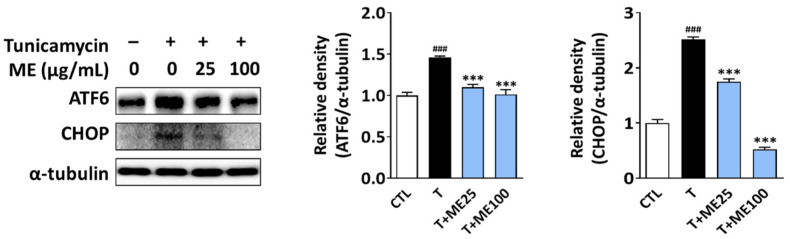
Effect of ME on ER stress signals. NIT-1 pancreatic β-cells were treated with ME alone (0, 25, or 100 μg/mL), followed by tunicamycin (0.05 μg/mL) co-treatment. The levels of ER stress-related proteins (ATF-6 and CHOP) were examined using Western blotting. Band density was quantified using ImageJ software. Results are represented as the mean ± SD of three separate experiments performed in triplicate. ### *p* < 0.001 versus CTL (vehicle-treated cells); *** *p* < 0.001 versus tunicamycin-treated cells. ROS: reactive oxygen species; T: tunicamycin; ME: Mori Ramulus ethanol extract.

Our data demonstrated that ME increased insulin secretion and ameliorated mitochondrial apoptosis in ER stressed cells. To gain further insight into the possible mechanism, we investigated whether ME could improve β-cell dysfunction via regulating ER stress signals. As shown in Figure 5, tunicamycin significantly induced ER stress as indicated by the significantly increased protein levels of ATF-6 and CHOP. Interestingly, there was a significant decrease in the protein levels of ATF-6 and CHOP in the tunicamycin- and ME-treated NIT-1 pancreatic β-cells compared to the tunicamycin-treated cells. These results indicate that ME may attenuate tunicamycin-induced ER stress.

### 3.5. Effect of ME on Body Weight, Organ Weight, and Serum Biochemical Profiles in Diabetic Mice

As shown in Table 1, HFD-fed mice showed an increase in body weight compared to that of ND-fed mice, but this alteration, as well as liver and adipose tissue weights, was not affected by ME administration (Table 1). Serum FFA levels were significantly decreased after ME administration. However, TC, HDL-C, and TG levels did not differ among the experimental groups (Table 2).

### 3.6. ME Improves Insulin Resistance and β-Cell Loss Induced by HFD Feeding

To investigate the effect of ME on hyperglycemia and hyperinsulinemia, we treated mice with an experimental diet containing ME for 15 weeks. As shown in Table 2, HFD-fed mice showed an increase in fasting blood glucose and insulin levels, whereas these levels were reduced by ME administration (−14.1%, −41.8%). Moreover, ME administration significantly decreased the HOMA-IR index by approximately two-fold, a marker of insulin resistance, compared to that of the HFD groups (*p* < 0.05).

**Figure 6:**
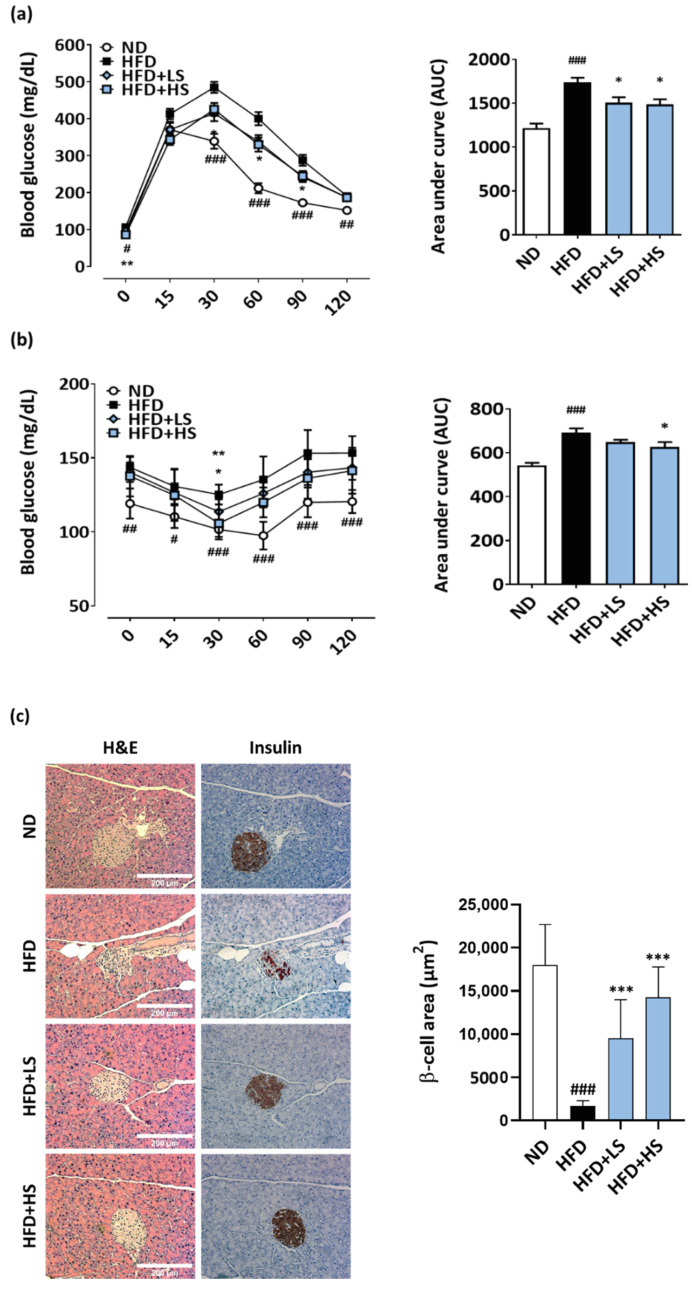
ME ameliorates hyperglycemia, glucose intolerance, and pancreatic β-cell mass loss in diabetic mice. (**a**) OGTT, (**b**) ipITT, and (**c**) representative images of H&E and immunohistochemistry for insulin staining (magnification 200×, scale bar = 200 μm). β-cell size was measured in insulin-stained sections of 8–10 islets from four mice per groups and was determined using the ImageJ software. Results are expressed as the mean ± SD (*n* = 8 per group). # *p* < 0.05, ## *p* < 0.01, ### *p* < 0.001 versus ND group; * *p* < 0.05, ** *p* < 0.01, *** *p* < 0.001 versus HFD group. OGTT, oral glucose tolerance test; ipITT, intraperitoneal insulin tolerance test; ND, normal diet; HFD, high-fat diet; HFD + LS, HFD plus 0.1% ME; HFD + HS, HFD plus 0.25% ME.

Next, to determine whether ME could improve insulin resistance induced by HFD, we performed OGTT and ipGTT. As shown in Figure 6a,b, ND-fed mice showed a normal response to glucose and insulin loading, whereas HFD-fed mice showed a markedly delayed glucose removal. However, ME administration effectively reduced the serum glucose levels. In addition, the areas under curve (AUC) for OGTT and ipITT were increased by 43.0% and 23.7%, respectively, in the HFD group compared to those in the ND group. However, the AUCs for OGTT and ipITT in the HFD + HS group decreased by 14.4% and 13.2%, respectively.

To investigate the effect of ME on β-cell function in vivo, we observed the islet morphology and β-cell mass using H&E and insulin immunohistochemical staining of pancreatic tissue sections. As shown in Figure 6c, the area of insulin-positive β-cells of the HFD-fed mice was smaller than that of the ND group. In contrast, the β-cell area of the HFD + LS- or HFD + HS-fed mice was similar to that of the ND group. Moreover, the β-cell area was significantly increased in HFD mice treated with ME, demonstrating that ME protects against β-cell loss in the diabetic state induced by HFD feeding.

### 3.7. ME Increased Hepatic Mitochondrial Enzyme Expression

To confirm the effect of ME on hepatic mitochondrial function in HFD-fed mice, hepatic mitochondria were isolated from mice in the four groups, and then the expression levels of mitochondrial enzymes and the functional aspect of the electron transport chain were measured based on their importance in metabolic health.

**Figure 7:**
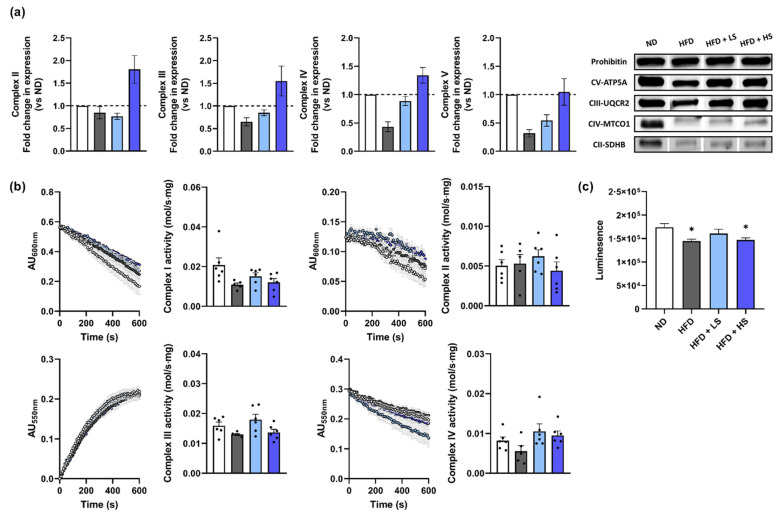
ME increased the expression level of complexes II–V in the liver of HFD-fed mice. (**a**) Normalized expression and immunoblot detection. (**b**) Kinetically monitored activity of complexes I–IV and calculated activity. (**c**) Mitochondrial ATP production. ND = white dots or bars, HFD = gray dots or bars, HFD + LS = light blue dots or bars, and HFD + HS = blue dots or bars. Results are expressed as the mean ± SEM. * *p* < 0.05 versus ND group. ND, normal diet; HFD, high-fat diet; HFD + LS, HFD plus 0.1% ME; HFD + HS, HFD plus 0.25% ME.

The pattern of fold change in the expression levels of mitochondrial complexes II–V showed a similar tendency between the groups (Figure 7a). All the complexes showed the highest expression level in the HFD + HS groups, followed by that of other experimental groups. This demonstrates that ME administration increased the expression level of hepatic mitochondrial complexes.

By using the same amount of mitochondrial protein, the enzymatic activities of mitochondrial complexes in the electron transport chain were kinetically measured using the spectral property of their specific reactant or product. From the linear portion of each progression curve, the specific activity of each complex was calculated based on Equation (1) and plotted (Figure 7b). Interestingly, no statistically significant differences were observed in the complex activity among the groups, however, the HFD group showed a slight decrease in its complex I, III, and IV activities compared with that of the ND group. This indicates that ME increased the expression level of mitochondrial complexes, but did not result in increased complex activity. This might have resulted from the effects of ME on the blood index and insulin resistance that forfended the over-activity of mitochondrial complexes that accompanied the increased ROS production in further steps. Accordingly, the ATP production levels of isolated mitochondria were similar in the HFD and HFD + HS groups, which were lower than those of the ND and HFD + LS groups (Figure 7c).

## 4. Discussion

ME has been used as a traditional medicine for T2DM treatment. Although previous studies have indicated that ME can function as a hypoglycemic and anti-obesogenic agent, the precise mechanism underlying its hypoglycemic action is not well understood. In this study, we described the protective effects of ME on apoptotic NIT-1 pancreatic β-cells of mice with HFD-induced T2DM by administrating 0.1% (HFD + LS group) and 0.25% ME (HFD + HS) with HFD. NIT-1 is an insulinoma cell line established from a transgenic NOD/Lt mouse [26]. Moreover, NIT-1 pancreatic β-cells are utilized in ER stress and T2DM as previously described [27,28,29]. Following ME treatment, tunicamycin-treated NIT-1 pancreatic β-cells with ER stress exhibited reduced apoptotic cell death, increased insulin secretion, reduced the expression of ER stress-related protein markers, decreased ROS production, maintained the ∆Ψm, reduced apoptotic pathway activation. Moreover, in HFD-induced diabetic animal model, ME supplementation prevented abnormal insulin and glucose response and pancreatic β-cell loss. Overall, our results, in addition to supporting previously determined ability of ME as a hypoglycemic agent, demonstrated the efficacy of ME on pancreatic β-cell function improvement, which was mediated by apoptotic pathway suppression and improved insulin resistance.

Previous studies have demonstrated that preserving the function of pancreatic β-cells prevents the occurrence of T2DM [30]. Similar to the ER stress inducer tunicamycin, a high-fat diet is also known to induce ER stress in several organs [31]. ME administration ameliorated diabetic conditions in HFD-fed mice, including HFD-induced ER stress, and inhibited ROS production and pancreatic β-cell apoptosis, which was consistent with our results in NIT-1 pancreatic β-cells and those of previous studies. Administration of ME along with HFD resulted in an ameliorated diabetic index including the levels of free-fatty acids, fasting glucose, insulin, and HOMA-IR index compared to that of the HFD group. Regarding the progression of T2DM in HFD mice, it appeared that insulin levels in the ME group were decreased, to levels similar to those in the ND group, compared to the HFD group. It demonstrates that ME efficiently increased insulin levels in the HFD group while also preventing β-cell failure and amelioration of insulin resistance [32]. In the diabetic condition, hyperglycemia and hyperlipidemia led the lipid partitioning toward a metabolized or esterified state leading to an inhibition of the insulin signaling pathway and fat accumulation in the liver [33]. ME successfully obstructed the progression of β-cell failure, and the metabolic health of the liver including insulin sensitivity was maintained in HFD groups supplemented with ME. In addition to the blood index, HFD feeding had a detrimental effect on hepatic mitochondrial function by decreasing the expression level and activity of mitochondrial complexes in the electron transport chain. However, ME administration increased the expression levels of hepatic mitochondrial complexes in a dose-responsive manner leading to higher expression levels in the HFD + HS group compared to the ND group. The overall complex activity and ATP production of the HFD + LS group were fully recovered to levels similar to that of the ND group. Earlier studies have indicated a disparity between enzyme expression and function with reduced activity or function even when the protein expression or mRNA level was increased. Furthermore, the enzymes that showed inconsistent expression and activity levels were mostly related to oxidative stress control [34,35]. In previous studies, hyper-activity of mitochondrial oxidative phosphorylation was shown to induce overproduction of ROS that led to ROS-induced apoptosis [36]. Consistent with a previous study, the hepatic mitochondrial function of the HFD + HS group might not surpass that of the ND group due to the maintenance of redox balance by preventing the over-activity of the mitochondrial enzymes and ameliorating diabetic indexes, including insulin resistance by ME supplementation. This represents a crosstalk between diabetic conditions and regulation of mitochondrial function in the liver as an insulin-sensitive organ. Although not all components of ME have been identified, we measured the content of oxyresveratrol and resveratrol (oxyresveratrol 385.84 μg/mg of the extract; resveratrol 20.73 μg/mg of the extract). Park et al. [37] reported that oxyresveratrol significantly increases insulin secretion in INS-1 cells and the level of insulin in plasma of diabetic mice. Jeon et al. [38] suggested that oxyresveratrol might be mainly responsible for the inhibition of α-glucosidase activity by the extract from ultraviolet C-irradiated mulberry leaves. In addition, oral administration of resveratrol ameliorated diabetic symptoms and improved insulin resistance in a streptozotocin-induced diabetic model [39]. Thus, we suggest the possibilities of synergistic interactions with other bioactive compounds in ME.

Taken together, our result suggested the efficacy of ME contributed to preserving the function of pancreatic β-cells that ameliorated T2DM. In addition, ME improved insulin sensitivity in the liver and increased the expression level of oxidative phosphorylation enzymes in hepatic mitochondria but did not alter the mitochondrial activity, which possibly prevented the production of ROS from the hyperactive enzymes in the electron transport chain. These results indicated a mechanistic role of ME on pancreatic β-cells, blood index, and hepatic mitochondria, and the cross talk between the organs demonstrates the role of ME as a hypoglycemic and anti-oxidant agent in depth.

## Figures and Tables

**Table 1:** Mouse body weight, food intake, and organ weight.

	ND	HFD	HFD + LS	HFD + HS
Initial body weight (g)	22.3 ± 1.2	22.2 ± 1.0	22.3 ± 1.2	22.3 ± 0.7
Final body weight (g)	34.1 ± 3.3	40.1 ± 4.4 ##	39.1 ± 3.2	38.5 ± 2.7
Body weight gain (g)	11.7 ± 2.3	17.8 ± 4.0 ##	16.7 ± 2.2	16.1 ± 3.0
Food intake (g/day)	2.4 ± 0.1	2.2 ± 0.1 #	2.2 ± 0.0	2.3 ± 0.1
Liver weight (g)	1.20 ± 0.32	1.46 ± 0.36	1.29 ± 0.19	1.34 ± 0.26
Epididymal fat weight (g)	1.64 ± 0.47	2.48 ± 0.50 ###	2.59 ± 0.27	2.42 ± 0.26
Retroperitoneal fat weight (g)	0.66 ± 0.20	1.03 ± 0.28 #	01.03 ± 0.21	0.90 ± 0.27

All values are expressed as the mean ± SD (*n* = 8 per group). # *p* < 0.05, ## *p* < 0.01, ### *p* < 0.001, were significant compared to the ND group; ND, normal diet; HFD, high-fat diet; HFD + LS, HFD plus 0.1% ME; HFD + HS, HFD plus 0.25% ME.

**Table 2:** Serum parameters in mice fed the experimental diet for 15 weeks.

	ND	HFD	HFD + LS	HFD + HS
Total cholesterol (mg/dL)	101.1 ± 22.3	123.6 ± 18.8	137.7 ± 11.4	132.0 ± 13.5
HDL cholesterol (mg/dL)	115.8 ± 26.9	146.7 ± 24.8 ##	166.2 ± 1.5	159.3 ± 11.5
Triglyceride (mg/dL)	93.6 ± 26.1	123.6 ± 18.6 #	116.4 ± 19.0	123.9 ± 18.5
Free fatty acid (μM/mL)	13.0 ± 2.3	21.1 ± 1.6 ###	17.5 ± 1.4 ***	17.2 ± 3.4 ***
Fasting glucose (mg/dL)	189.0 ± 6.3	235.2 ± 17.0 ###	230.7 ± 12.2	202.4 ± 10.0 ***
Insulin (ng/mL)	0.30 ± 0.12	1.65 ± 0.12 ###	1.33 ± 0.62	0.96 ± 0.50 *
HOMA-IR	4.05 ± 1.67	27.45 ± 3.25 ###	21.92 ± 10.54	13.97 ± 7.39 *

All values are expressed as mean ± SD (*n* = 8 per group). # *p* < 0.05, ## *p* < 0.01, ### *p* < 0.001, were significant compared to the ND group; * *p* < 0.05, *** *p* < 0.001, were significant compared to the HFD group; HOMA-IR = [fasting plasma glucose (mg/mL) × fasting plasma insulin (μU/mL)/405]; HFD + LS, HFD plus 0.1% ME; HFD + HS, HFD plus 0.25% ME.

## Data Availability

The data presented in this study are available on request from the corresponding authors.

## References

[1] Srinivasan K., Viswanad B., Asrat L., Kaul C.L., Ramarao P. (2005). Combination of high-fat diet-fed and low-dose streptozotocin-treated rat: A model for type 2 diabetes and pharmacological screening. Pharmacol. Res..

[2] Nadeau K.J., Zeitler P.S., Bauer T.A., Brown M.S., Dorosz J.L., Draznin B., Reusch J.E., Regensteiner J.G. (2009). Insulin resistance in adolescents with type 2 diabetes is associated with impaired exercise capacity. J. Clin. Endocrinol. Metab..

[3] Shulman G.I. (2014). Ectopic fat in insulin resistance, dyslipidemia, and cardiometabolic disease. N. Engl. J. Med..

[4] Cree-Green M., Triolo T.M., Nadeau K.J. (2013). Etiology of insulin resistance in youth with type 2 diabetes. Curr. Diab. Rep..

[5] Lee K.U., Harris R.A. (2012). Mitochondria and endoplasmic reticulum in diabetes and its complications. Exp. Diabetes Res..

[6] Di Martino R., Sticco L., Luini A. (2019). Regulation of cargo export and sorting at the trans-Golgi network. FEBS Lett..

[7] Ma Z.A., Zhao Z., Turk J. (2012). Mitochondrial dysfunction and beta-cell failure in type 2 diabetes mellitus. Exp. Diabetes Res..

[8] Ristow M., Zarse K., Oberbach A., Kloting N., Birringer M., Kiehntopf M., Stumvoll M., Kahn C.R., Bluher M. (2009). Antioxidants prevent health-promoting effects of physical exercise in humans. Proc. Natl. Acad. Sci. USA.

[9] Loh K., Deng H., Fukushima A., Cai X., Boivin B., Galic S., Bruce C., Shields B.J., Skiba B., Ooms L.M. (2009). Reactive oxygen species enhance insulin sensitivity. Cell Metab..

[10] Orrenius S. (2004). Mitochondrial regulation of apoptotic cell death. Toxicol. Lett..

[11] Butler A.E., Janson J., Bonner-Weir S., Ritzel R., Rizza R.A., Butler P.C. (2003). Beta-cell deficit and increased beta-cell apoptosis in humans with type 2 diabetes. Diabetes.

[12] Meeprom A., Chan C.B., Sompong W., Adisakwattana S. (2018). Isoferulic acid attenuates methylglyoxal-induced apoptosis in INS-1 rat pancreatic beta-cell through mitochondrial survival pathways and increasing glyoxalase-1 activity. Biomed. Pharmacother..

[13] Krentz A.J., Bailey C.J. (2005). Oral antidiabetic agents: Current role in type 2 diabetes mellitus. Drugs.

[14] Prattichizzo F., La Sala L., Ryden L., Marx N., Ferrini M., Valensi P., Ceriello A. (2019). Glucose-lowering therapies in patients with type 2 diabetes and cardiovascular diseases. Eur. J. Prev. Cardiol..

[15] Miyazaki Y., Glass L., Triplitt C., Wajcberg E., Mandarino L.J., DeFronzo R.A. (2002). Abdominal fat distribution and peripheral and hepatic insulin resistance in type 2 diabetes mellitus. Am. J. Physiol. Endocrinol. Metab..

[16] Wang S., Fang M., Ma Y.L., Zhang Y.Q. (2014). Preparation of the Branch Bark Ethanol Extract in Mulberry Morus alba, Its Antioxidation, and Antihyperglycemic Activity In Vivo. Evid. Based Complement. Alternat. Med..

[17] Yin X.L., Liu H.Y., Zhang Y.Q. (2017). Mulberry branch bark powder significantly improves hyperglycemia and regulates insulin secretion in type II diabetic mice. Food Nutr. Res..

[18] Ahn E., Lee J., Jeon Y.H., Choi S.W., Kim E. (2017). Anti-diabetic effects of mulberry (Morus alba L.) branches and oxyresveratrol in streptozotocin-induced diabetic mice. Food Sci. Biotechnol..

[19] Ham I.J.E., Lee B., Choi H. (2008). The Study on Anti-hypertensive and Anti-diabetic Effect of Mori Ramulus. Kor. J. Herbol..

[20] Park Y.H., An M., Kim J.K., Lim Y.H. (2020). Antiobesity effect of ethanolic extract of Ramulus mori in differentiated 3T3-L1 adipocytes and high-fat diet-induced obese mice. J. Ethnopharmacol..

[21] Yoshida S., Ohishi T., Matsui T., Tanaka H., Oshima H., Yonetoku Y., Shibasaki M. (2011). The role of small molecule GPR119 agonist, AS1535907, in glucose-stimulated insulin secretion and pancreatic beta-cell function. Diabetes Obes. Metab..

[22] Ko E., Um M.Y., Choi M., Han T., Kim I.H., Shin S. (2020). Cassia tora Seed Improves Pancreatic Mitochondrial Function Leading to Recovery of Glucose Metabolism. Am. J. Chin. Med..

[23] Choi W.H., Ahn J., Jung C.H., Jang Y.J., Ha T.Y. (2016). Beta-Lapachone Prevents Diet-Induced Obesity by Increasing Energy Expenditure and Stimulating the Browning of White Adipose Tissue via Downregulation of miR-382 Expression. Diabetes.

[24] Marre M.L., James E.A., Piganelli J.D. (2015). Beta cell ER stress and the implications for immunogenicity in type 1 diabetes. Front. Cell Dev. Biol..

[25] Guha P., Kaptan E., Gade P., Kalvakolanu D.V., Ahmed H. (2017). Tunicamycin induced endoplasmic reticulum stress promotes apoptosis of prostate cancer cells by activating mTORC1. Oncotarget.

[26] Hamaguchi K., Gaskins H.R., Leiter E.H. (1991). NIT-1, a pancreatic beta-cell line established from a transgenic NOD/Lt mouse. Diabetes.

[27] Jung T.W., Lee M.W., Lee Y.J., Kim S.M. (2012). Metformin prevents endoplasmic reticulum stress-induced apoptosis through AMPK-PI3K-c-Jun NH2 pathway. Biochem. Biophys. Res. Commun..

[28] Eizirik D.L., Cardozo A.K., Cnop M. (2008). The role for endoplasmic reticulum stress in diabetes mellitus. Endocr. Rev..

[29] Lee H., Im S.W., Jung C.H., Jang Y.J., Ha T.Y., Ahn J. (2016). Tyrosol, an olive oil polyphenol, inhibits ER stress-induced apoptosis in pancreatic beta-cell through JNK signaling. Biochem. Biophys. Res. Commun..

[30] Buchanan T.A., Xiang A.H., Peters R.K., Kjos S.L., Marroquin A., Goico J., Ochoa C., Tan S., Berkowitz K., Hodis H.N. (2002). Preservation of pancreatic beta-cell function and prevention of type 2 diabetes by pharmacological treatment of insulin resistance in high-risk hispanic women. Diabetes.

[31] Castro G., MF C.A., Weissmann L., Quaresma P.G., Katashima C.K., Saad M.J., Prada P.O. (2013). Diet-induced obesity induces endoplasmic reticulum stress and insulin resistance in the amygdala of rats. FEBS Open Bio.

[32] Kasuga M. (2006). Insulin resistance and pancreatic beta cell failure. J. Clin. Investig..

[33] Muoio D.M., Newgard C.B. (2008). Mechanisms of disease:Molecular and metabolic mechanisms of insulin resistance and beta-cell failure in type 2 diabetes. Nat. Rev. Mol. Cell Biol..

[34] Omar R.A., Chyan Y.J., Andorn A.C., Poeggeler B., Robakis N.K., Pappolla M.A. (1999). Increased Expression but Reduced Activity of Antioxidant Enzymes in Alzheimer’s Disease. J. Alzheimers Dis..

[35] Wauthier V., Schenten V., Verbeeck R.K., Calderon P.B. (2006). Ageing is associated with increased expression but decreased activity of CYP2E1 in male Wistar rats. Life Sci..

[36] Huttemann M., Helling S., Sanderson T.H., Sinkler C., Samavati L., Mahapatra G., Varughese A., Lu G., Liu J., Ramzan R. (2012). Regulation of mitochondrial respiration and apoptosis through cell signaling: Cytochrome c oxidase and cytochrome c in ischemia/reperfusion injury and inflammation. Biochim. Biophys. Acta.

[37] Park S.Y., Jin B., Shin J.H., Adisakwattana S., Kwon O. (2017). Standardized Mori ramulus extract improves insulin secretion and insulin sensitivity in C57BLKS/J db/db mice and INS-1 cells. Biomed. Pharmacother..

[38] Jeon Y.H., Choi S.W. (2019). Isolation, Identification, and Quantification of Tyrosinase and alpha-Glucosidase Inhibitors from UVC-Irradiated Mulberry (Morus alba L.) Leaves. Prev. Nutr. Food Sci..

[39] Su H.C., Hung L.M., Chen J.K. (2006). Resveratrol, a red wine antioxidant, possesses an insulin-like effect in streptozotocin-induced diabetic rats. Am. J. Physiol. Endocrinol. Metab..

